# Second reply to the second letter to the editor by Alessandria, Berrino, Malatesta and Donzelli (doi: 10.17179/excli2026-9442)

**DOI:** 10.17179/excli2026-9443

**Published:** 2026-04-15

**Authors:** Lamberto Manzoli, Cecilia Acuti Martellucci, Maria Elena Flacco

**Affiliations:** 1 Department of Medical and Surgical Sciences, University of Bologna, 40100 Bologna, Italy; 2 Department of Environmental and Prevention Sciences, University of Ferrara, 44121 Ferrara, Italy

## ⁯⁯⁯⁯

We appreciate the effort to establish a constructive scientific debate by Alessandria, Berrino, Malatesta and Donzelli (Alessandria et al., 2026[3]).

We had extensively acknowledged the biases inherent to our studies in the primary text (Acuti Martellucci et al., 2025[1]; Flacco et al., 2023[5]), and these authors tried to quantify further bias using the following methods: (1) unadjusted estimation of death rates (Berrino et al., 2023[4]), and (2) calculation of death hazard ratios without adjusting for age, in a sample where the vaccinated individuals were 8 years older, on average, than the unvaccinated (Alessandria et al., 2024[2]). We clarified our study design in the previous reply (Manzoli et al., 2025[6]), however we fear that the dialogue may not develop further if Alessandria et al. defend the major methodological faults described above on the basis of one report which did use multivariable models adjusted for age (Weberpals et al., 2018[8]), and another one which conducted Monte-Carlo simulations adjusted for randomly generated baseline covariates (Mi et al., 2016[7]).

Given the above, we believe that the only possible conclusion is that, whatever the bias was in our study, it was smaller than the biases of the studies that were supposed to disprove our findings.

## Declaration

### Conflict of interest

All authors declare no conflict of interest.

### Artificial Intelligence (AI) – assisted technology

No artificial intelligence was used.
